# Genital mycoplasma infection: a systematic review and meta-analysis

**DOI:** 10.1186/s12978-023-01684-y

**Published:** 2023-09-12

**Authors:** Chen Cheng, Xiangyu Chen, Yuxuan Song, Shangren Wang, Yang Pan, Shuai Niu, Rui Wang, Li Liu, Xiaoqiang Liu

**Affiliations:** 1 Charcteristic Medical Center of the Chinese People’s Armed Police Force, Tianjin, 300162 China; 2 Department of Urology, Tianjin Medical University General Hospital, 154 Anshan Road, Heping District, Tianjin, 300052 China; 3 Department of Urology, Peking University People’s Hospital, Beijing, 100044 China

**Keywords:** Genital mycoplasma, Infection, Male infertility, Systematic review, Meta-analysis

## Abstract

**Background:**

Recent studies have suggested that genital mycoplasma infections may be associated with male infertility. However, this association remains controversial due to time lapse, sample size, and regional prevalence.

**Objectives:**

This study aimed to systematically evaluate the relationship between genital mycoplasma and male infertility through a meta-analysis and to provide a basis for the clinical management of male infertility.

**Methods:**

We conducted a search on PubMed, EMBASE, the Cochrane Library, and CNKI databases, from January 2000 to June 2023 to identify case–control studies on the interrelationship between genital mycoplasma infection and male infertility. Two independent researchers performed an assessment of the methodological quality of trials according to the Newcastle–Ottawa scale and extracted data strictly based on the inclusion and exclusion criteria, and afterward, we carried out a meta-analysis using Stata 16.0. Pooled odds ratios (OR) with 95% confidence intervals (CI) were used to assess this relationship.

**Results:**

This meta-analysis included 21 studies from seven countries with a total of 53025 infertility cases and 6435 controls; the age range of the participating men was from 20 to 59 years old. The results obtained showed a higher prevalence of *M. genitalium*, *M. hominis* and *U. urealyticum* infections in infertile men than in the controls, with the opposite result for *U. parvum* (*M. genitalium*, OR, 3.438 [95% CI: 1.780, 6.643], with P = 0.000; *M. hominis*, OR, 1.840 [95% CI: 1.013, 3.343], with P = 0.045; *U. urealyticum*, OR, 3.278 [95% CI: 2.075, 5.180], with P = 0.000; *U. parvum*, OR, 1.671 [95% CI: 0.947, 2.950], with P = 0.077). Further, two subgroup analyses also showed that *M. hominis* and *U. urealyticum* infections were strongly associated with male infertility in China (*M. hominis*, P = 0.009; *U. urealyticum*, P = 0.000); however, *M. hominis* and *U. urealyticum* infection was not strongly associated with male infertility worldwide (*M. hominis*, P = 0.553; *U. urealyticum*, P = 0.050).

**Conclusion:**

This meta-analysis revealed that male infertility was significantly associated with *M. genitalium*, *M. hominis* and *U. urealyticum* infections, while *U. parvum* infection was not*.* Further, our study showed that genital mycoplasma infection influences male infertility and provides a basis for future treatment.

## Introduction

Male infertility is defined as the inability of a male to become pregnant with a fertile female after at least one year of unprotected intercourse [1]. According to the World Health Organization, approximately 15% of couples of reproductive age worldwide have fertility problems, with the male factor accounting for approximately 50% of them [2]. The risk factors contributing to male infertility include lifestyle (drug, alcohol, smoking, and weight etc.), environmental (pesticides and organic solvents, heavy metals like lead, and radiation exposure) and medical conditions (infection, tumor, retrograde ejaculate, surgeries, anti-sperm antibodies, undescended testicles, chromosomal defects, and hormone imbalances etc.) [3–6]. Reproductive tract infections can cause leukocytosis of semen, the release of inflammatory factors, and altered levels of reactive oxygen species (ROS). They may also lead to secondary vas deferens obstruction, resulting in infertility. Among the many pathogens, although *Mycoplasma spp.* and *Ureaplasma spp.* contain bacteria that are natural inhabitants of male urethra, are potentially pathogenic species that play an etiologic role in genital infections and male infertility [7]. However, based on the available data, there is no conclusive evidence on reproductive mycoplasma infections that show their association with male fertility and the pathways through which they elicit their effects.

There are two pathogens of the *Mycoplasma spp.* that are especially relevant to andrology: *Mycoplasma genitalium* and *Mycoplasma hominis*. And andrologically important *Mycoplasma spp.* are *Mycoplasma hominis* and *Mycoplasma genitalium*, both of which are causing urogenital infections [8–10]. The prevalence of *Mycoplasma spp.* infection in the general population is 1.3% in developed countries and 3.9% in developing countries [11]. However, the patients infected with *M. genitalium* tend to be asymptomatic in their clinical presentation [12]. Only less than 10% of men in the general population with *Mycoplasma spp.* infections are symptomatic in terms of difficulty urinating, increased penile irritation, urethritis, and infertility. Early in vitro studies found that *Mycoplasma spp.* infection reduced sperm agglutination and viability, as well as damaged sperm DNA resulting in reduced sperm counts [7]. Because there are no significant clinical symptoms after infection with *Mycoplasma spp.*, patients often ignore relevant laboratory tests and miss the best opportunity for treatment.

In 2001, Robertson proposed the division of *Ureaplasma urealyticum* into *Ureaplasma parvum*, while the original Cluster A was retained as *Ureaplasma urealyticum* [13]. The prevalence of *U. urealyticum* ranges from 10 to 40% and is thought to be associated with prostatitis, epididymitis, and male infertility [14]. In several studies, the detection rate of *U. urealyticum* in the semen of infertile men (5–58%) was higher than that of fertile men (3–31%) [15, 16]. Since *U. urealyticum* plays a dual role, reducing sperm motility at low pH and increasing sperm velocity at high pH, the mechanism by which *U. urealyticum* causes infertility remains unclear [17]. And in many studies, *U. parvum* and *U. urealyticum* have not been studied separately.

Although there is a growing body of research on *U. urealyticum*, there is still very little research on *M. hominis* and *M. genitalium*, and in particular, there are few large-sample studies of semen testing in people infected with *M. genitalium*. Furthermore, the effects of *M. hominis* and *M. genitalium* on male fertility have not been clearly described. Therefore, this study aimed to provide an evidence-based clinical practice approach through a systematic evaluation of clinical literature on the levels of genital mycoplasma infection in infertile and fertile men between January 2000 and June 2023, with the intention of providing insight into the association between *U. urealyticum*, *M. hominis*, and *M. genitalium* infections and male infertility.

## Methods

We conducted this review to examine whether genital mycoplasma infection is associated with male infertility by comparing the number of infertility cases in the genital mycoplasma infection group with the number of fertility cases in the control group.

### Study selection

**Fig. 1:**
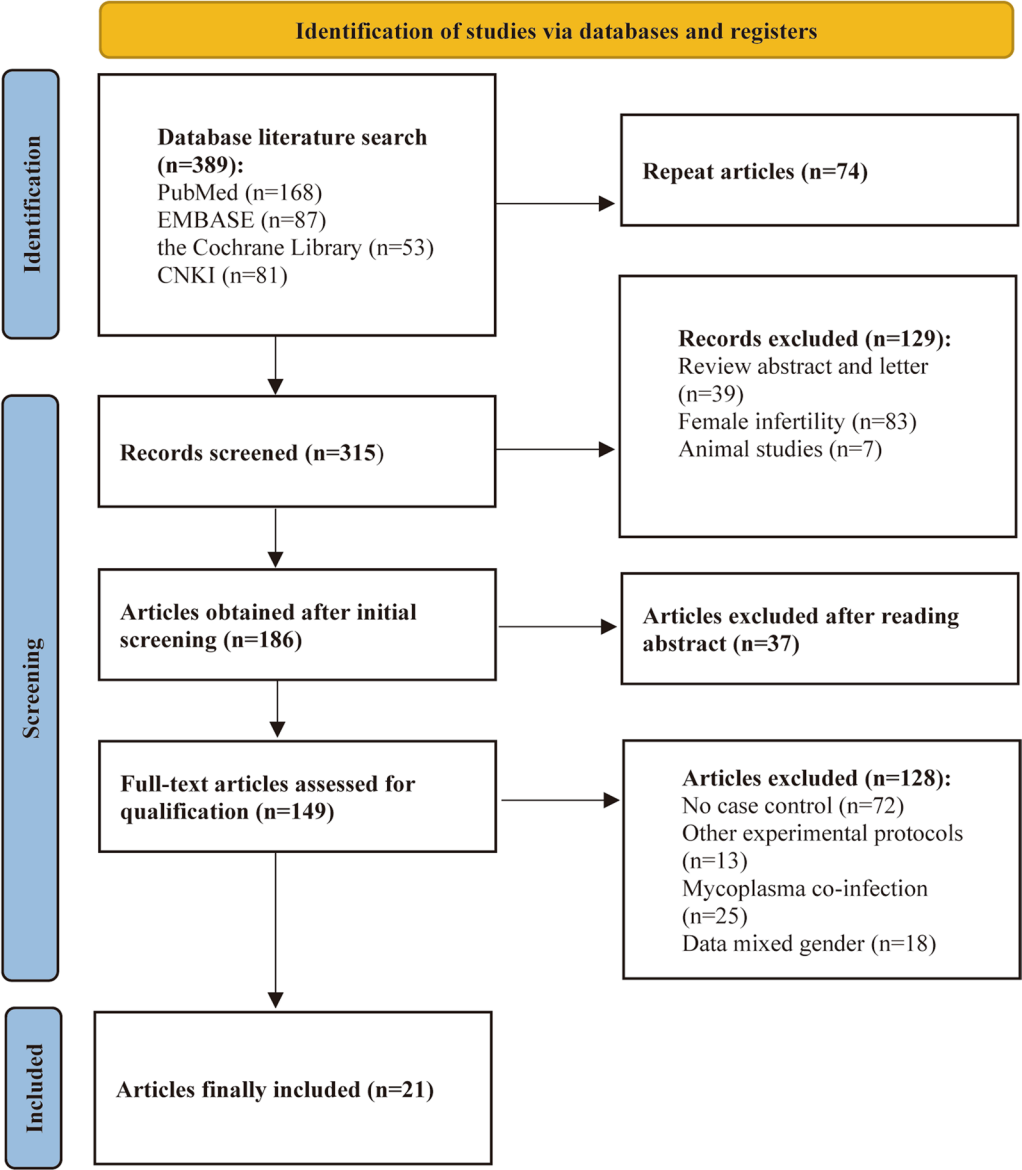
Flow chart for PRISMA-based literature screening

This systematic review and meta-analysis were conducted based on the PRISMA statement [18]. According to the PRISMA flow diagram (Fig. 1), we searched PubMed, EMBASE, the Cochrane Library, and CNKI databases from January 2000 to June 2023, using text and keywords in combination, both as MeSH (Medical Subject Headings) terms and text words. Search terms used in the search strategy: “*Mycoplasma*,” “*Mycoplasma genitalium*,” “*Mycoplasma hominis*,” “*Ureaplasma urealyticum*,” “*U. parvum*,” “Infertility,’ “Sterility,” and “Semen quality.” For articles with overlapping data and the same authors, the most recent or comprehensive study was used, and for the same author or duplicate data from different journals, the most recent or comprehensive study was used. Review articles, letters, commentaries, case reports, and preclinical studies were excluded.

### Inclusion and exclusion criteria

Eligible studies were included based on the following inclusion criteria: (a) the types of studies included in the literature were all *M. genitalium*, *M. hominis*, *U. urealyticum*, and *U. parvum* case–control studies; (b) the experimental specimens were from infertile men, and the control specimens were from normal fertile men; (c) the test specimens were from male semen, prostatic fluid, or urethral secretions; (d) studies containing sufficient data to allow for calculation of a pooled odds ratio (OR) and 95% confidence interval (95% CI). The exclusion criteria were as follows: (a) duplicate publications or poor quality information; (b) insufficient primary data and fruitless requests or incomplete study data; (c) reviews, abstracts, commentaries, etc.; (d) infertility or fertility groups where mixed gender and data could not be separated; and (e) animal studies.

### Data extraction

Screening and data extraction was carried out by two researchers who first conducted literature screening and data extraction independently, according to the criteria developed. The reasons for excluding articles were also recorded. When a disagreement arises, both parties negotiate or consult with a third-party expert. The established literature screening criteria were as follows: initial screening to exclude articles that clearly did not meet the criteria based on the title and abstract, followed by a detailed reading of the literature to select the final articles for inclusion in this study based on the inclusion and exclusion criteria. The following data were collected from the included articles: first author, country of study, year of publication, sample size, detection methods, and specimen source.

### Assessment of study quality

The quality of the included studies was assessed using the Newcastle–Ottawa scale. The scale consists of eight items covering three dimensions: (a) selection of study groups (4 points), (b) comparability of groups (2 points), and (c) ascertainment of exposure and outcomes (3 points) for the case–control. The scale assigns a maximum score of nine, which represents a high-quality study [19].

### Statistical analysis

The data were processed using Stata 16.0 software. To calculate the relationship between genital mycoplasma and male infertility, the OR was used as an indicator of effect, and the pooled OR and 95% CI were calculated. A P-value of < 0.05 was considered to be statistically significant. Heterogeneity testing of the literature was performed using Higgins’ I^2^. If I^2^ is < 50%, multiple similar studies would be considered homogeneous, and a fixed-effects model would be used for the analysis. On the other hand, if I^2^ is > 50%, multiple similar studies would be considered heterogeneous and combined using a random-effects model. We observed publication bias by plotting funnel plots, and Begg’s rank correlation was used to assess potential publication bias. After which, we then conduct a subgroup analysis of the prevalence between regions.

## Results

### Characteristics of included studies

The systematic search of the four databases yielded 389 articles. 74 duplicate articles retrieved from different databases were first excluded and, upon further examination, a further 294 articles were excluded based on the inclusion and exclusion criteria established previously, leaving a total of 21 articles. There were 53025 primary and secondary infertility cases and 6435 controls in the 21 studies conducted in seven countries, including China, Korea, Iran, Kuwait, Jordan, Denmark, and Estonia [20–40]. All included studies had been scored based on Newcastle–Ottawa Case–Control Study Scale (Table 1). The data extracted from each study were qualitatively synthesized and are presented in Table 1. All the subjects in these 21 studies were tested or treated at hospitals. Specimens were collected from semen, urethral swabs, or first void urine (FVU) and then tested by polymerase chain reaction (PCR), culture, SAT, or mycoplasma IST. 7 studies focused on *M. genitalium* infections, comprising 29859 cases and 950 controls. 9 studies were related to *M. hominis* infection, containing 21,305 cases and 4783 controls. There were 18 studies on *Ureaplasma spp.* infections, including 14 studies on *U. urealyticum* and 4 studies on *U. parvum*, making a total of 23,113 cases and 5536 controls.

**Table 1:** Characteristics of the case–control studies included in the meta-analysis

Species	First author	Year	Country	Age range	Method of detection	Specimen	Case	Control	Newcastle–Ottawa Scale
*M. genitalium*	Tjagur	2021	Estonia	N/A	PCR	Semen samples	22/2000	0/248	6
	Ahmadi	2018	Iran	24–59	PCR	Semen samples	16/165	2/165	7
	Li	2018	China	N/A	SAT	Semen samples	651/27314	1/200	7
	Safavifar	2015	Iran	N/A	PCR	Semen samples	6/15	11/30	6
	Plecko	2014	Denmark	N/A	Culture and PCR	FVU	2/145	0/49	7
	Abusarah	2013	Jordan	20–58	PCR	FVU and semen samples	3/93	1/70	8
	Al-Sweih	2012	Kuwait	N/A	PCR	Semen samples	4/127	1/188	6
*M. hominis*	Ahmadi	2016	Iran	30–41	PCR	Semen samples	24/165	6/165	7
	Huang	2015	China	20–44	Culture	Urethral swab	604/19098	37/3368	6
	Liu	2014	China	N/A	Culture	Semen samples	36/621	30/615	5
	Plecko	2014	Denmark	N/A	Culture and PCR	FVU	31/145	10/49	7
	Zhang	2014	China	22–36	Culture	Semen samples and Urethral swab	40/815	2/158	6
	Lee	2013	Korea	N/A	Culture	Semen samples	7/50	3/48	6
	Wang	2013	China	24–41	Culture	Urethral swab	4/176	1/150	6
	Zeng	2013	China	N/A	Culture	Urethral swab	6/108	0/42	5
	Al-Sweih	2012	Kuwait	N/A	PCR	Semen samples	10/127	33/188	6
*U. urealyticum*	Zhou	2017	China	21–45	Culture	Semen samples	56/540	10/260	6
	Ma	2016	China	24–42	Culture	Semen samples	12/37	0/12	7
	Huang	2015	China	20–44	Mycoplasma IST	Semen samples	1951/19098	123/3368	8
	Liu	2014	China	N/A	Culture	Semen samples	165/621	153/615	6
	Zhang	2014	China	23–48	Culture and PCR	Semen samples	32/223	8/146	6
	Zhang	2014	China	22–36	Culture	Semen samples and Urethral swab	183/815	15/158	5
	Abusarah	2013	Jordan	20–58	PCR	FVU and semen samples	1/93	2/70	6
	Lee	2013	Korea	N/A	Culture	Semen samples	24/50	12/48	7
	Wang	2013	China	24–41	Culture	Urethral swab	70/176	9/150	8
	Al-Sweih	2012	Kuwait	N/A	PCR	Semen samples	17/127	24/188	7
	Zhang	2011	China	23–41	Culture	Semen samples	156/967	0/201	6
	Zeng	2011	China	N/A	Culture and PCR	Semen samples	32/120	3/120	8
	Zeighami	2009	Iran	21–50	PCR	Semen samples	12/100	3/100	7
	Najar Peerayeh	2008	Iran	21–50	PCR	Semen samples	23/146	3/100	7
*U. parvum*	Zhou	2017	China	21–45	Culture	Semen samples	127/540	32/260	6
	Zhang	2014	China	23–48	Culture and PCR	Semen samples	43/223	28/146	8
	Abusarah	2013	Jordan	20–58	PCR	FVU and semen samples	9/93	2/70	7
	Zeighami	2009	Iran	21–50	PCR	Semen samples	3/100	2/100	6

*N/A* not available

### Correlation between *M. genitalium* infection and male infertility

**Fig. 2:**
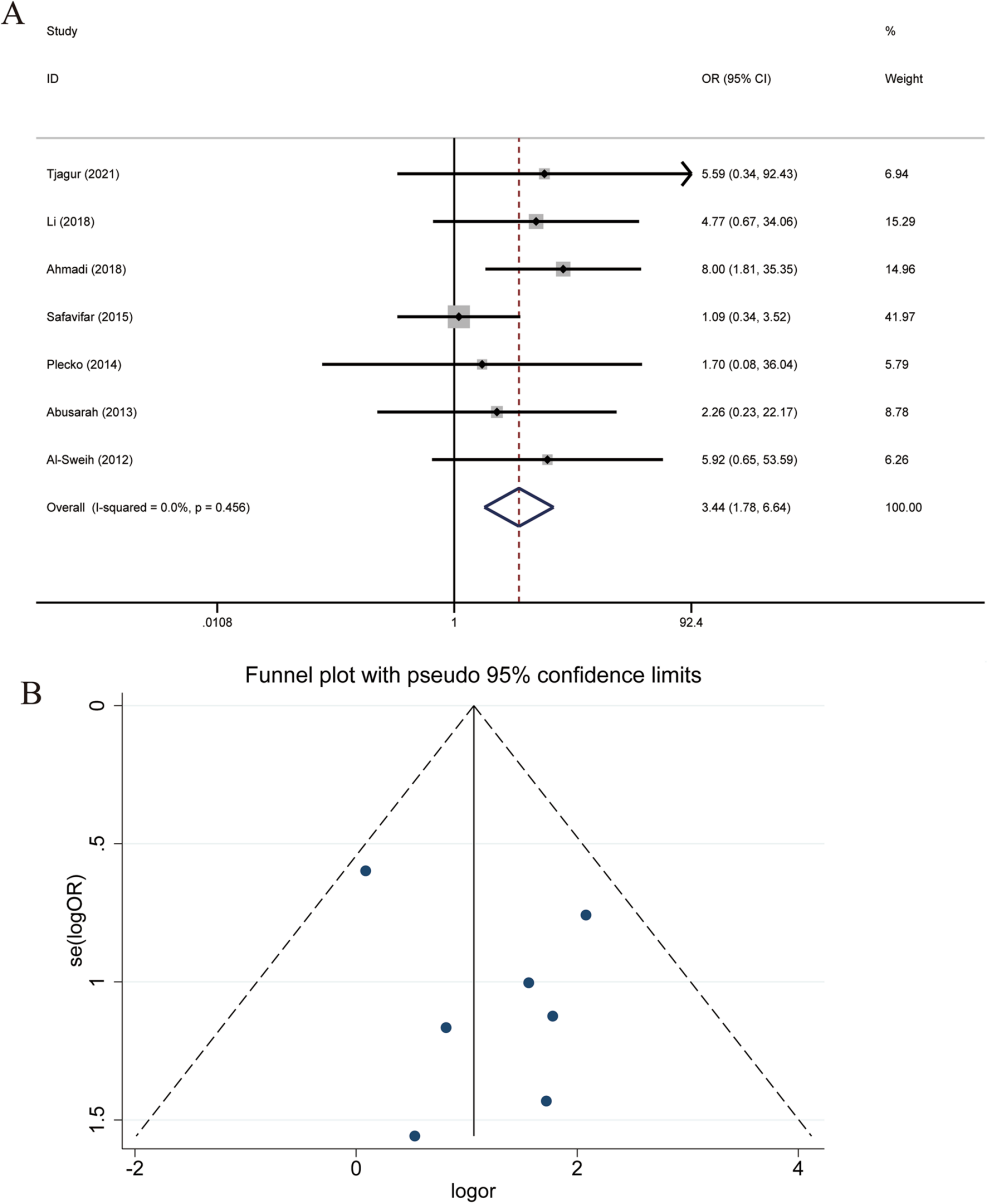
**A** Forest plot for the association of *M. genitalium* infection and male infertility. **B** Funnel plots for inclusion in studies of *M. genitalium* infection and male infertility

The analysis of the case and control groups for each study outcome regarding *M. genitalium* infection revealed no statistical heterogeneity among the studies regarding the population with *M. genitalium* infection, X^2^ = 5.72, df = 6, P = 0.456, and I^2^ = 0.0%. The pooled OR of all included studies was 3.438(95% CI: 1.780, 6.643), with Z = 3.68 and P = 0.000 (Fig. 2A), suggesting a statistically significant association between *M. genitalium* and male infertility.

To detect publication bias, a funnel plot analysis was performed on the included studies (Fig. 2B). Because the symmetry of funnel plots is subjective by observation, Begg’s test and Egger’s test correlation were used to quantify the funnel plots for a more objective evaluation. In Begg’s test, P = 1.000, and in Egger’s test, P = 0.165, suggesting that there may not be a large publication bias.

### Correlation between *M. hominis* infection and male infertility

**Fig. 3:**
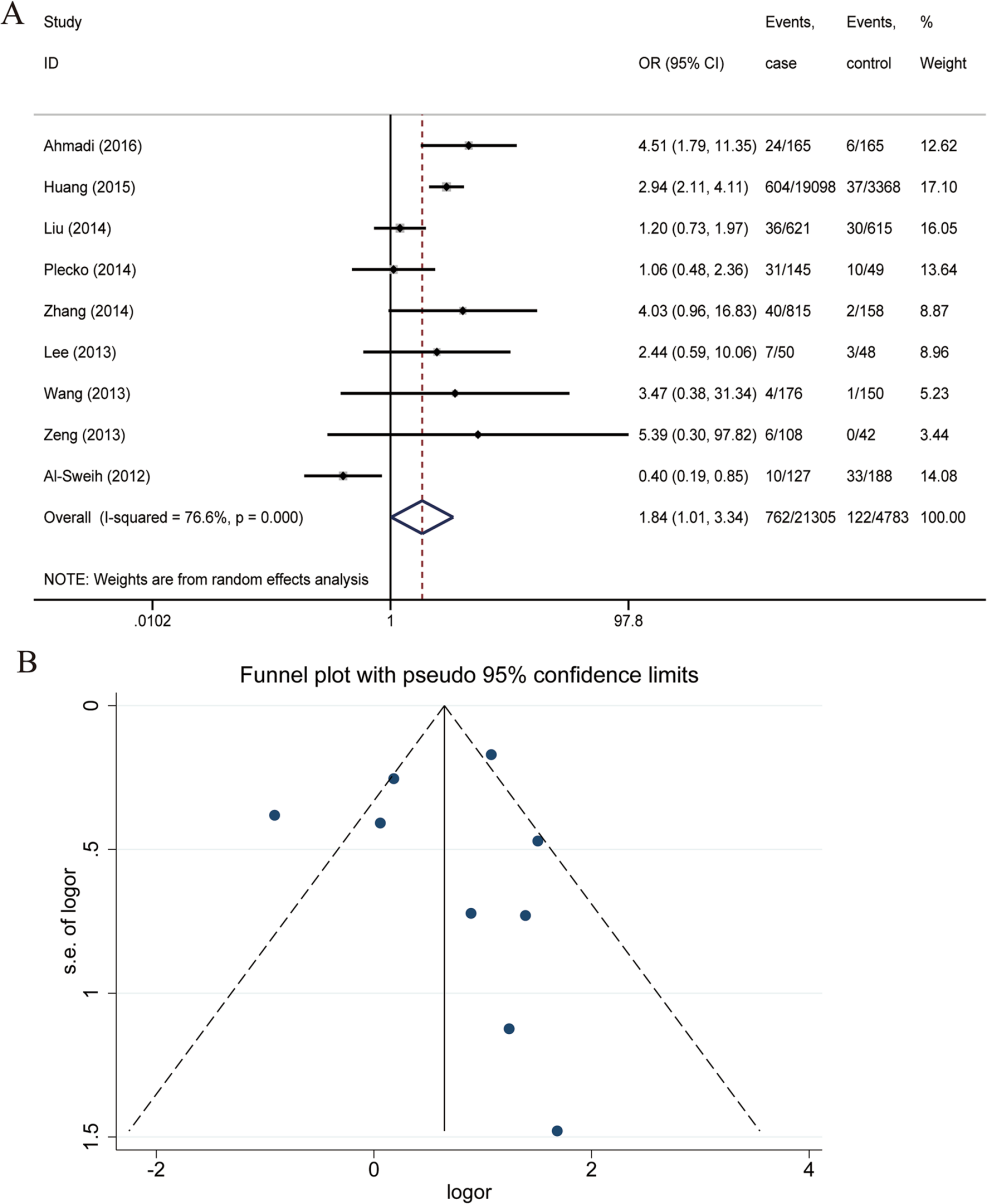
**A** Forest plot for the association of *M. hominis* infection and male infertility. **B** Funnel plots for inclusion in studies of *M. hominis* infection and male infertility

The analysis of the case and control groups for each study outcome regarding *M. hominis* infection revealed that there is a statistical heterogeneity among the studies regarding the population with *M. hominis* infection, X^2^ = 34.25, df = 8, P = 0.000, I^2^ = 76.6%. The pooled OR of all included studies was 1.840 (95% CI: 1.013, 3.343), with Z = 2.00 and P = 0.045 (Fig. 3A), which suggests a statistically significant association between *M. hominis* infection and male infertility.

The participants were then divided into a world group and a Chinese group according to their geographical regions for subgroup analysis. The pooled OR of the world group was 1.399 (95% CI: 0.462, 4.241), with Z = 0.59 and P = 0.553, suggesting that there is no statistically significant association between *M. hominis* and male infertility worldwide. The pooled OR of the Chinese group was 2.323 (95% CI: 1.238, 4.358), with Z = 2.63 and P = 0.009, suggesting a statistically significant association between *M. hominis* and male infertility in China. Sensitivity analysis was performed because of the high heterogeneity. The Chinese subgroup was found to be significantly less heterogeneous after the exclusion of one study by removing studies one by one; at this point, P = 0.951, I^2^ = 0.0%. Sensitivity analysis of the world subgroup revealed good stability of the results and no reduction in heterogeneity. In this study, Begg’s test gave a P-value of 0.602, and Egger’s test gave a P-value of 0.907, suggesting that there may not be a large publication bias (Fig. 3B).

### Correlation between *U. urealyticum* infection and male infertility

**Fig. 4:**
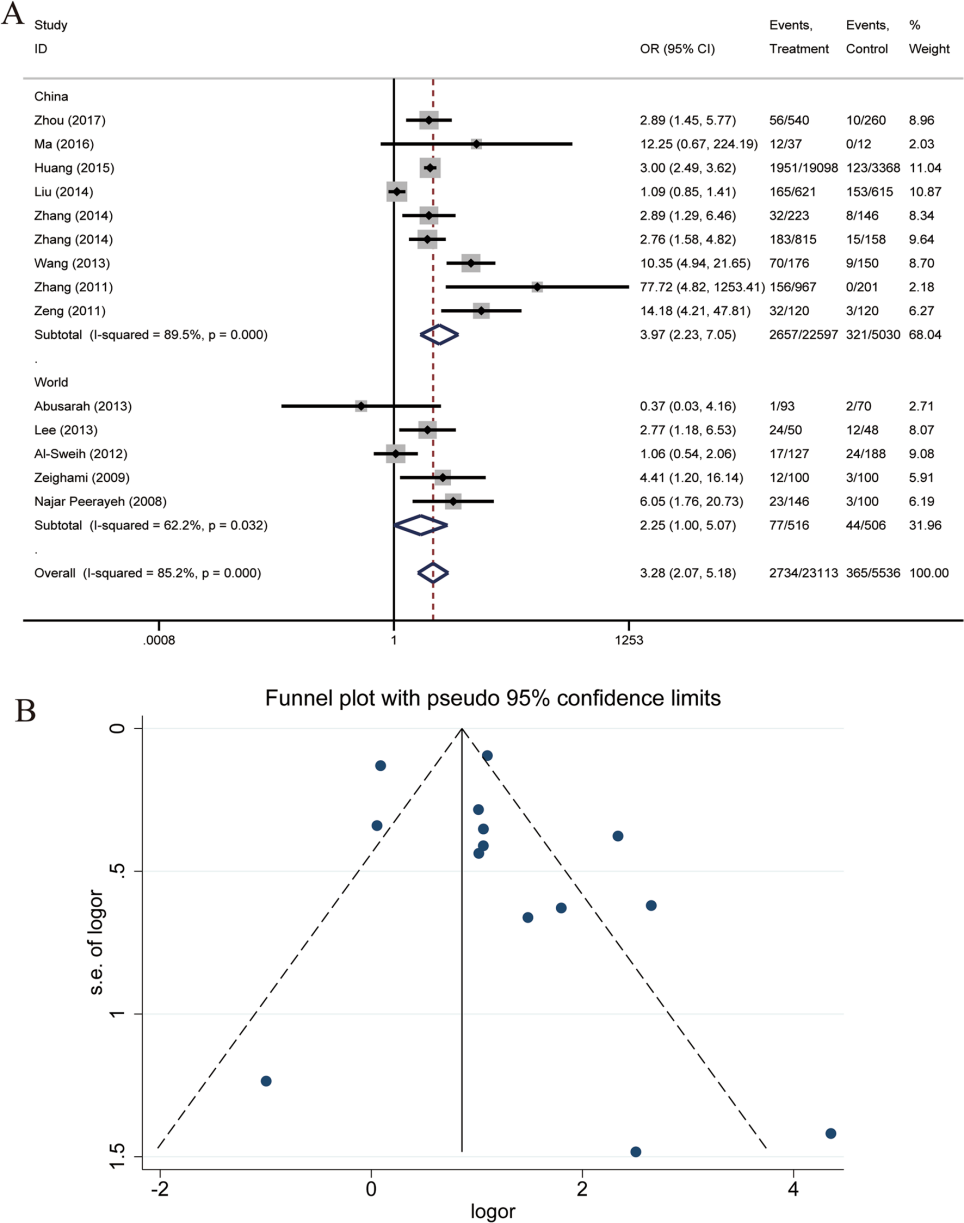
**A** Forest plot for the association of *U. urealyticum* infection and male infertility. **B** Funnel plots for inclusion in studies of *U. urealyticum* infection and male infertility

The analysis of the case and control groups for each study outcome regarding *U. urealyticum* infection revealed that there is a statistical heterogeneity among the studies regarding the population with *U. urealyticum* infection, X^2^ = 87.91, df = 13, P = 0.000, I^2^ = 85.2%. The pooled OR of all included studies was 3.278 (95% CI: 2.075, 5.180), with Z = 5.09 and P = 0.000 (Fig. 4A), suggesting a significant association between *U. urealyticum* and male infertility.

The participants were also divided into a world group and a Chinese group. The pooled OR of the world group was 2.250 (95% CI: 0.999, 5.069), with P = 0.050, suggesting no statistically significant association between *U. urealyticum* and male infertility in the world. The pooled OR of the Chinese group was 3.967 (95% CI: 2.232, 7.050), with P = 0.000, suggesting a statistically significant association between *U. urealyticum* and male infertility in China. Sensitivity analysis of both subgroups showed no significant reduction in heterogeneity. In this study, Begg’s test gave a P-value of 0.584, and Egger’s test gave a P-value of 0.252, suggesting that there may not be a large publication bias (Fig. 4B).

### Correlation between *U. parvum* infection and male infertility

**Fig. 5:**
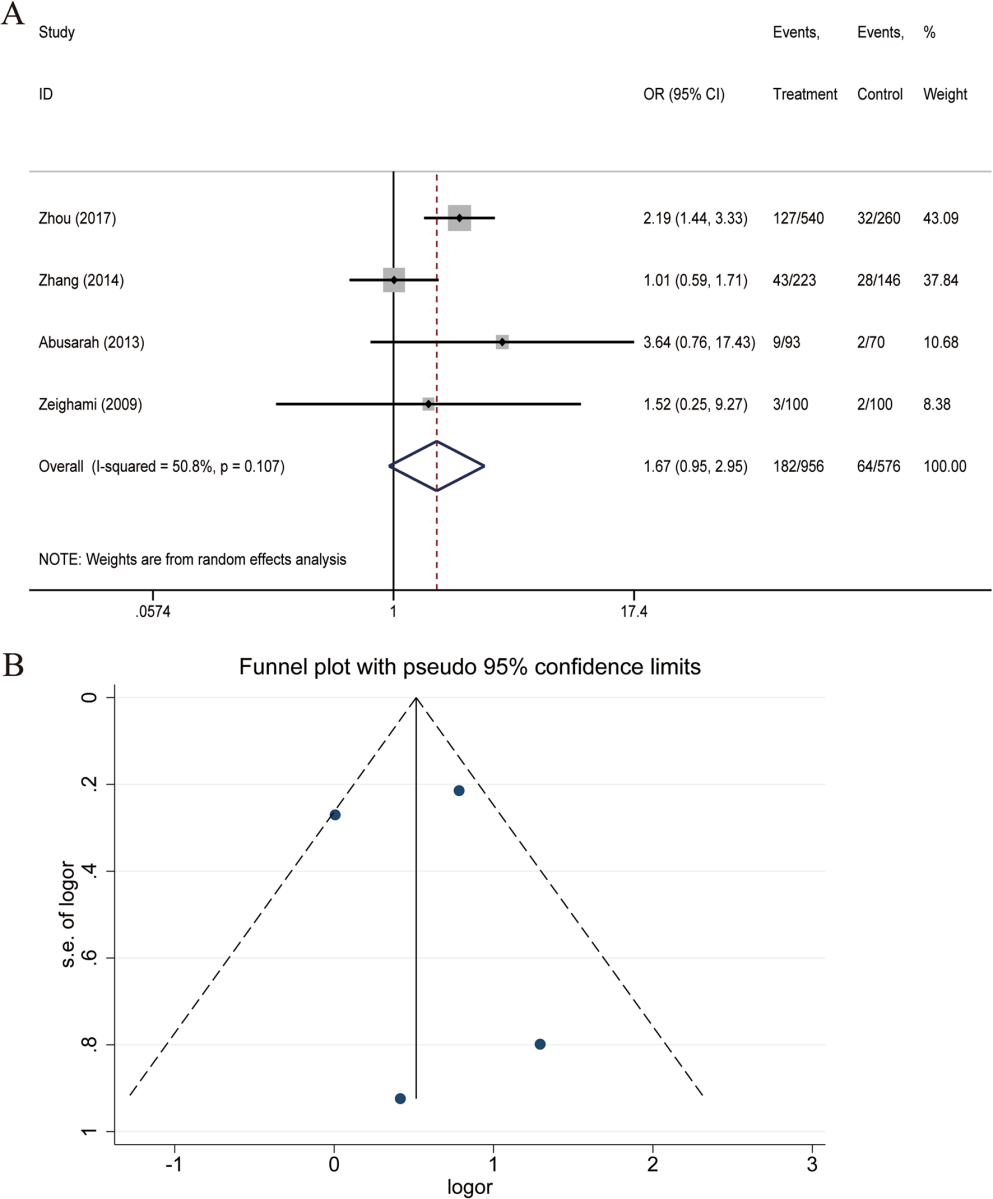
**A** Forest plot for the association of *U. parvum* infection and male infertility. **B** Funnel plots for inclusion in studies of *U. parvum* infection and male infertility

The analysis of the case and control groups for each study outcome regarding *U. parvum* infection revealed no statistical heterogeneity among the studies regarding the population with *U. parvum* infection, X^2^ = 6.10, df = 3, P = 0.107, I^2^ = 50.8%. The pooled OR of all included studies was 1.671 (95% CI: 0.947, 2.950), with Z = 1.77 and P = 0.077 (Fig. 5A), suggesting no statistically significant association between *U. parvum* and male infertility. In this study, Begg’s test gave a P-value of 0.734, and Egger’s test gave a P-value of 0.902, suggesting that there may not be a large publication bias (Fig. 5B).

## Discussion

According to the results, our meta-analysis found that male infertility was significantly correlated with *M. genitalium*, *M. hominis* and *U. urealyticum*. Moreover, the infertile men had more *M. genitalium, M. hominis* and *U. urealyticum* infections than fertile men. However, male infertility is not significantly associated with *U. parvum*.

The meta-analysis incorporated the most recent studies and expanded the study population. Based on our results, we drew conclusions that differ from previous studies and provided further evidence to show the relationship between genital mycoplasma infection and male infertility [41]. In contrast to our results, that previous meta-analysis reported that *U. urealyticum* and *M. hominis* were significantly associated with male infertility, but *U. parvum* and *M. genitalium* were not. We analyzed the possible reasons for the different results, and in this study we expanded the sample size of the study and, following our search criteria, we included eight additional articles published in recent years after that meta-analysis. At the same time, we also took into account the expansion of the study area, which helped to reduce regional or ethnographic effects from a single area. What’s more, we performed a subgroup analysis and considered possible sources of heterogeneity through sensitivity analysis. In addition differences in specimen source and detection methods for genital mycoplasma infections may also contribute to differences in results, with higher detection rates of mycoplasma in semen than urethral swabs in asymptomatic patients with mycoplasma infections. Moreover, the sensitivity of SAT was higher than that of PCR and culture among the detection methods for mycoplasma infection, compared to our meta-analysis, which included more studies in which the source of the specimen was sperm and the detection method was SAT [42].

In the subgroup analysis of *M. hominis* infection, the results of the world group showed that *M. hominis* infections were not associated with male infertility, and sensitivity analysis could not exclude studies that affected heterogeneity. However, the Chinese group showed significantly lower heterogeneity after excluding one article [27], so it is possible that the grouping basis and statistical approach in those articles contributed to the high heterogeneity of the studies. Also, in the study conducted on *U. urealyticum* infection, the exclusion of any of the literature does not reduce the heterogeneity of the study, so the sources of heterogeneity may still be due to the grouping basis.

Studies have shown that approximately 15% of male infertility is currently associated with genital infections, and that damage caused by mycoplasma such as non-gonococcal urethritis (NGU), orchitis, and prostatitis tends to be moderate compared to the severe symptoms caused by Chlamydia trachomatis and gonococcus in the reproductive tract, including testicular atrophy and obstructive azoospermia [43–45]. Genital mycoplasma is closely associated with male genitourinary infections, and NGU is the most common disease of the genital tract in men [46]. *M. hominis* and *U. urealyticum* can commensally exist in the urethra, due to their low inflammatory characteristics, most patients are asymptomatic [47]. Antibiotics are the main treatment for genital mycoplasma infections, and recent studies have shown that moxifloxacin is an effective treatment for *M. genitalium*, but antimicrobial resistance limits its oral treatment options [48]. Azithromycin may be used instead of moxifloxacin if macrolide susceptibility can be ascertained using molecular resistance tests [49]. And so far, there has been considerable disagreement on the exact association of *M. genitalium* with male infertility [50]. Similarly, the evidence for *Ureaplasma spp.* as pathogens causing infertility is less conclusive than that for Chlamydia trachomatis and Neisseria gonorrhoeae [51]. The results of our study indicate that urogenital infections caused by *M. genitalium*, *M. hominis* and *U. urealyticum* are correlated with male infertility, which provide new basis for further revealing the relationship between urogenital mycoplasmas and male infertility.

Laboratory diagnosis of genital mycoplasma is important in the prevention of infertility. Culture approach to detect bacterial infections have shown good sensitivity and specificity, but nucleic acid amplification tests have significantly higher sensitivity for asymptomatic infections with low organisms’ load, which is a significantly relevant advantage [52]. Multiplex assays of PCR offer an option for patients with pathogen co-infection, this technologies are available for the detection of up to eighteen different organisms, and their sensitivity and specificity are comparable with their respective singleplex assays, which help solve the problem of *U. urealyticum* and *U. parvum* that cannot be diferentiated in culture [53]. Another test for the identification of genital mycoplasma (*M. genitalium, M. hominis* and *U. urealyticum*) showed consistent results with PCR on clinical samples, but with higher sensitivity at lower DNA concentrations [54]. These more economical and convenient methods facilitate the spread of mycoplasma diagnosis.

This meta-analysis has some limitations. First, the literature studies were sourced from only seven countries, most of which were from China and the Middle East, with only three studies from Europe, and no relevant studies were reported from the Americas; thus making it impossible to determine whether there are environmental or geographical differences in the content of our study [55, 56]. Second, risk factors for male infertility vary over time and there have been a few case–control studies on mycoplasma infections and male infertility in recent years [57], therefore, more recent studies are needed to determine whether this correlation is influenced by temporal changes. Third, the detection of co-infections with pathogens that may cause male infertility, which was not part of our study, was also not represented in some studies [7, 58].

Currently, many patients with genital mycoplasma infections have mild symptoms and variability in the samples collected for evaluation, which may increase the risk of infertility if left undiagnosed and untreated for long periods of time [59]. Despite some limitations, this meta-analysis provides some evidence for the clinical management of male infertility due to genital tract mycoplasma infections. We also expect future statistical analyses of genital mycoplasma collection methods to determine optimal samples and sampling sites [60]. A more extensive study of mycoplasma globally will help to better understand its epidemiology and pathogenesis and to develop appropriate strategies for the treatment and prevention of male infertility.

## Conclusions

In summary, our meta-analysis suggested *M. genitalium*, *M. hominis* and *U. urealyticum*, but not *U. parvum*, were associated with male infertility. We looked forward to larger sample sizes of different ethnic populations required to confirm our findings. In addition, in further studies, an assessment of *U. parvum* is needed to demonstrate the association with male infertility.

## Data Availability

The datasets employed in the current study can be available from the corresponding author upon the reasonable request.
